# A model of farmers intentions towards organic farming: A case study on rice farming in Thailand

**DOI:** 10.1016/j.heliyon.2019.e03039

**Published:** 2019-12-28

**Authors:** Phaibun Yanakittkul, Chuenjit Aungvaravong

**Affiliations:** a Royal Irrigation Department, Bangkok, Thailand; b Faculty of Business Administration and Accountancy, Khonkaen University, Thailand

**Keywords:** Business, Agriculture, IOF model, Organic farming behaviour, Policy support, Comparison of farmers' intentions

## Abstract

Determining the factors that influence intentions towards organic farming (IOF) is a challenge. This research applied the theory of planned behaviour to determine factors which influence farmers’. There are six causal factors: attitudes towards farming behaviour, group-norm influences on farming behaviour, perceived behavioural control of farmers, comparative usefulness of behaviours, perceived of risk of farming and support of government policy. The purpose of this article is to explore the influential factors for conserving and cumulating organic farming behaviours, which are compared between organic and conventional rice farmers. The result provides government agencies an outline of how to increase organic farming, especially for smallholder farmers, and the long-term benefits will decrease poisonous contamination and increase human health.

## 1 Introduction

Farming is a critical and essential food-production process for humanity. By the year 2050, the world's population will grow to 9.1 billion (United Nations [UN], 2015). The agricultural-sector should increase food production by 70% to nourish the world's population. This increase should consist of 3 billion tonnes of grain and 0.47 billion tonnes of meat (Food and Agriculture Organization of the United Nations [FAO], 2015). The farming changes should include the use of chemical fertilisers to accelerate growth (Chakrabarty et al., 2014). For this reason, more than 5 million tonnes of agricultural chemicals will be used annually by the year 2000 (Fernando, 2017). Therefore, the large annual use of pesticides will accumulate and contaminate the ecosystem.

The effects of agricultural chemical contamination in the air, water, and soil profoundly affect human health through the accumulation of toxins from living in an environment full of toxins and the consumption of toxic food (Katherine and Hendrik, 2010). This effect should increase the rates of asthma, autism, physical disabilities, learning disabilities, reproductive disorders, diabetes, Parkinson disease, Alzheimer disease, and cancer (Owens et al., 2010). In addition to directly affecting human health, chemicals from agricultural activities also affect the ecosystems of plants and animals. Finally, humans are affected by the consumption of these products and meat (Onder et al., 2011; Sharma and Singhvi, 2017). The use of chemical fertilisers, insecticides, and pesticides has continued for a long time in the agricultural production process and is widely found in farm systems and small farms (Aktar et al., 2009; Savci, 2012), where the health effects are found both in consumers and farmers who use chemicals for agricultural activities (Costa et al., 2014).

Consequently, avoiding the use of chemicals by substituting current practices with organic farming is an appropriate solution to these human and environmental problems (Sharma and Singhvi, 2017). The consumption of organic products is increasing because of the awareness of the dangers of contaminated foodstuffs (Sangkumchaliang and Huang, 2012). Organic food is gaining popularity among consumers who love the earth and want to care for their health. FiBL and IFOAM (2016) have reported that the organic market increased to US$ 80 billion in 2014 mainly because of economic activity driven by developed countries such as the United States and those in Western Europe. Accordingly, organic products are a new trend and a great opportunity for manufacturers in the food industry. The total area of organic farming was 43.7 million hectares (0.99% of the world's arable land). The integration of environmental protection and economic opportunity (Ferella et al., 2019) lead to sustainable agriculture; first, recycled organic waste will increase organic soil matter (Ulm et al., 2019); second, increasing organic farmers' sustainable behaviour will decrease poisonous contamination, which benefits human health (Yanakittkul and Aungvaravong, 2017). The expected results from the model of farmers' intentions towards organic farming (IOF) will suggest how extended knowledge of the theory of planned behaviour (TPB) can be applied by government agencies to increase organic farming, especially for smallholder farmers, and that the long-term benefit will decrease poisonous contamination and improve human health.

Therefore, the urgent need to promote and set a policy to enlarge organic farming is critical. The focus of this research is to find elements that influence farmer's behaviour towards organic rice farming in Thailand. In Thailand, rice is a critical economic crop and exports approximately 11 million tonnes of rice, accounting for 26% of the world's total rice exports (United States Department of Agriculture, 2015). The export value is US$ 5.37 billion. The major export markets for rice are China, Hong Kong, Singapore, Iran, Iraq, European, the United States, Canada, the Netherlands, Italy and France (Thai Rice Exporters Association, 2015).

## 2 Conceptual framework

The two main types of organic agriculture research are related to organic food and organic farmers. First, many studies have examined consumer behaviour and the consumer acceptance of organic products, the attitudes and perceptions of the benefits of organic foods (Lee and Yun, 2015), the effects of the price of organic products on consumer purchasing behaviours (Rödiger and Hamm, 2015), and the adoption of organic food consumption in Europe (Aarset et al., 2004). These studies have demonstrated that consumers are concerned about the health benefits and nutritional value of organic products (Schleenbecker and Hamm, 2013) and the behaviours and beliefs that influence organic food consumption have been examined (Zagata, 2012). Second, studies of organic farmers, which are of interest to this study, have investigated organic farming practices that are safe for farmers' health (Costa et al., 2014); the positive effects on farmer income, safety, and environmental sustainability (Setboonsarng, 2006); the attitudes and behaviour of farmers regarding organic farming (Power et al., 2013); and farmers’ behavioural changes after working on organic farms (Sutherland and Darnhofer, 2012).

Various management theories have been applied to study the behaviour of farmers such as: 1) Theory of planned behavior (TPB), van Dijk et al. (2016), Lalani et al. (2016); van Dijk et al. (2016), Sok et al. (2016); Borges et al. (2016), 2) Protection Motivation Theory (PMT), Dang et al. (2014), 3) Diffusion of Innovation Theory (DIT), Aubert et al. (2012). Based on the aforementioned theory, Krueger et al. (2000) and; Läpple and Kelley (2013) have indicated that the TPB is appropriate for the study of organic farming behaviour. The TPB considers three factors, namely, attitude to behaviour, subjective norm influence, and perceived behaviour control, which influence the intention towards behaviours.

The related factors that have been studied which influence farmers’ IOF are appropriate for this research. First, the factors from the TPB presented by Ajzen (1991) are three factors that affect intention to engage in a behaviour.

1) **Attitudes towards farming behaviour** refer to the concept that a farmer who has a positive attitude towards farming behaviour will intend to perform that behaviour. Borges, Tauer, and Lansink (2016) found that the attitudes increased farmers' intention to use improved natural grassland [effect size 0.46]. Likewise, Lalani et al. (2016) found that farmers' attitudes towards using conservation agriculture showed the highest effect size at 0.593. Furthermore, many studies have confirmed the attitudes of farmers regarding certain behaviours through various case studies, such as Jones et al. (2016), effect size 0.497; van Dijk et al. (2016), effect size 0.17; Sok et al. (2016), effect size 0.61; and Deng et al. (2016), effect size 0.327.
2) **Group–norm influences on farming behaviour** refer to the group-norm influencing farmer intention towards farming behaviour. For example, if a farmer perceives reference group-norm behaviour as good, the behaviour will be encouraged. Deng et al. (2016) found that farmers influenced by the subjective norm intended to pay for an ecosystem service programmer with an effect size of 0.418. Likewise, Jones et al. (2016) found that the subjective norm influences farmers intention to improve herd health with an effect size of 0.495. In addition, many studies have confirmed that the reference of individual/group-norm affects farmers' behaviour, including Lalani et al. (2016), effect size 0.155; van Dijk et al. (2016), effect size 0.11; Sok et al. (2016), effect size 0.18; Borges et al. (2016), effect size 0.237; and Dang et al. (2014), effect size 0.118.
3) **Perceived behaviour control towards farming behaviour** is used to evaluate the ability of a farmer to perform a behaviour that they are capable of controlling with intention towards farming behaviour. Therefore, many research studies have examined the perceived behaviour control of farmers such as Jones et al. (2016), who found that the perceived behaviour control of farmers improves herd health with an effect size of 0.523. Lalani et al. (2016) found that the perceived ability of smallholder farmers to control their behaviour affected their conservation agriculture behaviour with an effect size of 0.341. Moreover, Deng et al. (2016), van Dijk et al. (2016), and Borges et al. (2016) confirmed that the perceived ability to control farmer behaviour affected their behaviour. By contrast, Sok et al. (2016) found that perceived behavioural control did not significantly affect the behaviours of farmers to design voluntary bluetongue vaccination strategies.

In addition, some factors that have been applied to study the intention of farming behaviour such as the factors that follow.

1) **Perceptions of the risk of farming** is an external factor for a farmer's consciousness of the risks of farming and affects the behaviour of farmers. Yazdanpanah, et al. (2014) found that the perception risk of farmers affected their intentions and behaviours regarding water conservation with an effect size of 0.14. Likewise, Dang et al. (2014) found that farmers' perception risk of climate change affected productivity, financial status, and the health of agriculture with an effect size of 0.155. Similarly, Niles et al. (2013) found that farmer's perceptions and responses affected climate policy risks with an effect size of 0.72. Furthermore, Barnes et al. (2013) suggested that 62% of farmers in the European Union (EU) remain uncertain of the effects of climate change, with only 20% acknowledging the effect of climate change. In addition, Lehmann et al. (2013) found that climate and price risks slightly affected land use change and plant selection because of other factors such as soil type, technological constraints, and labour. The perception risk of market demand and agricultural crop prices are factors that determine behavioural change for variety of crops (Hardaker et al., 2015).
2) **Support of government policy** is based on government or private sector policy and must incentivise farmers to comply or behave in accordance with that policy. Morone et al. (2019) demonstrated that the policies (public food waste rule, investments and infrastructure, small scale farming) are drivers of the sustainable food consumption model. Dang et al. (2014) studied the variable of policy incentives to promote the cultivation of a variety of plants, and a policy to support the purchase of productive hedgerows; farmer intention was not affected. Likewise, Giannoccaro and Berbel (2013) found that a policy of agricultural subsidies did not affect farmer intentions to reduce the use of chemical fertilizers or water in agricultural activities. By contrast, Tate et al. (2012) found that government and local council support provided assistance in the use of renewable energy for farmers, with an effect size of 0.014, and bank loans to invest in energy applications, with an effect size of 0.016.
3) **Self–identify towards farming behaviour** is a concept in which the farmers discover what is suitable for them. van Dijk et al. (2016) identified farmers as individuals who preserve the environment and found an influence on the intention to not accept subsidies, with an effect size of 0.52. However, Yazdanpanah et al. (2014) investigated the self-identify of farmers with regard to water conservation and found no concerns about water conservation or the importance of participating in conservation activities.

Furthermore, the application of innovation diffusion theory (Rogers, 2003) which five factors. **First, comparative usefulness of behaviour**, is a factor that farmers will adopt and apply towards farming behaviour if benefits increase productivity. Aubert, Schroeder, and Grimaudo (2012) found that the benefits of applying technology in soil analysis and the appropriate type of plants affect farmers' perceived benefits and lead to applications of technology; Previously, Sattler and Nagel (2010) found that farmers accept different environmental measures by comparing the benefits in terms of risk cost and time required. Warren, Burton, Buchanan, and Birnie (2016) found that farmers accepted alternative energy crops because of their recognition of the long-term benefits. **Second, compatibility of behaviour** is a factor that farmers will accept and adopt if consistent with application. Sattler and Nagel (2010) found that farmers agree that different environmental measures are consistent with climate and land-farm characteristics. In addition, Warren et al. (2016) found that farmers did not accept alternative energy crops because they are inconsistent and incompatible with planting on the farm. Likewise, Aubert et al. (2012) showed that farmers applied technology because of compatibility with analyses of soil and plant species. **Third**, **complexity of behaviour** is farmers’ willingness to accept and adopt something because it is not difficult and not too complicated for them to use. Warren et al. (2016) found that farmers accepted alternative energy crops because they were easy to grow on farms. In the same manner, Sattler and Nagel (2010) found simple and easy-to-teach techniques for farm workers that farmers adopted under different environmental measures. **Fourth, trialability of behaviour** is a factor in which farmers accept and adopt needs to be practised. Sattler and Nagel (2010) found that farmers could experiment with technology in small spaces available on older computers. **Finally, observeability of behaviour** is a factor that farmers will accept and adopt behaviours through the observation of results. Sattler and Nagel (2010) found that farmers accept different environmental measures because of the image of the farm in society and positive environmental effects. Aubert et al. (2012) found that farmers applied technology to analyse soil and plant species grown based on observations from other individuals and to confirm the results.

**Figure 1:**
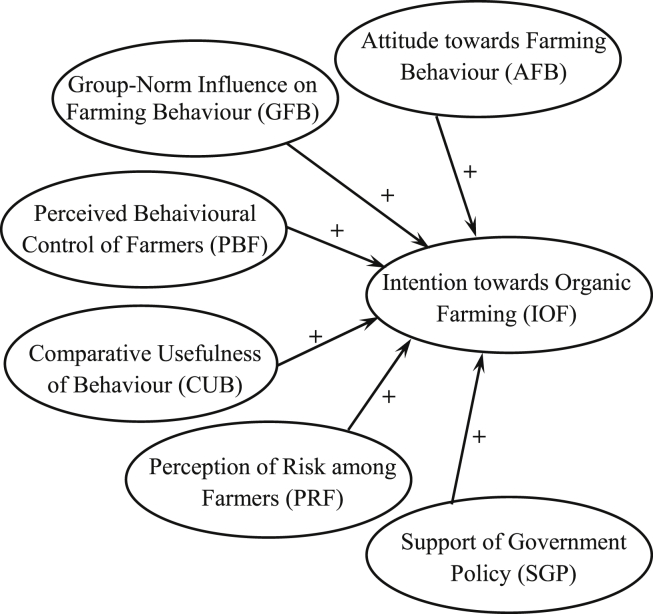
Conceptaul Framework: Model of farmers' intentions towards organic farming (Applied from Theory of planned behaviour, Ajzen, 1991).

The purpose of this article is to compare organic and conventional rice farmers, and especially an exploration of the influential factors for conserving and cumulating organic farming behaviours. In the literature, this could only be by intentions towards behaviour because conventional rice farmers had not yet grown organic rice. Therefore, the researcher application TPB model in with factors influencing intention toward behaviour. From above factors, were tested them on a focus group composed of organic rice farmers and conventional rice farmers. It was found that six factors should be used to test causal relationship with regard to intentions towards organic farming (IOF) as follows; 1) Attitudes towards farming behaviour (AFB); 2) Group–norm influences on farming behaviour (GFB); 3) Perceived behavioural control of farmers (PBF); 4) Comparative usefulness of behaviour (CUB); 5) Perceptions of the risk of farming (PRF); and 6) Support of government policy (SGP), see Figure 1.

## 3 Methodology

The researcher developed a questionnaire based on the application of Läpple and Kelley (2013), Yazdanpanah et al. (2014), Dang et al. (2014), van Dijk et al. (2016), Deng et al. (2016), Chang et al. (2016), Tate et al. (2012), Borges et al. (2016), Aubert et al. (2012), Sattler and Nagel (2010), and Yanakittkul and Aungvaravong (2017). Next, questionnaire was pre-tested using a focus group comprising eight organic rice farmers and seven conventional rice farmers. Finally, the questionnaire comprised of 37 items and scale questions divided into five levels: (1) very low, (2) low, (3) moderate, (4) high, and (5) very high (Appendix A). The structural equation model (SEM) was used in the analysis to compare organic and conventional rice farmers and explore the influential factors for conserving and cumulating organic farming behaviours. The sample size necessary for the SEM provides an appropiate to goodness of fit for the model. Criteria for determining the sample size is critical for SEM because if the sample is too small, the estimation increase the effect size (beta-coefficient, β; Tabacnick and Fidell, 2007). Therefore, the sample size must be sufficiently large so it will result in small tolerances and consistent estimation of parameters (Snijders and Bosker, 1999). Kline (2011) discusses the appropriateness of sample size, that is, it should higher than 200 samples. In addition, Kahai and Cooper (2003) and Hair et al. (2010) have defined sample size based on parameters and mention using ten samples for one observed variable. As a result, this study used the rule of ten samples per one observed variable; thus, the sample size should be higher than 370 for both organic and conventional rice farmers.

The sample group assignments are in accordance with the research objectives that compare the intention towards organic rice behaivoiur preferences of the two sample groups.

1) Group of organic rice farmers. Thailand had 19 groups which received an organic rice certifcate: 1,711 members and an area for organic rice of 13,277 ha (ACT Certificated Organic Operators, 2016). This research required a sample with strong groups that greater than 100 members and a certificate from IFOAM, EU or COR standards. Five groups fulfilled condition, for a total 1,045 member farmers (Table 1).

**Table 1:** The samples divided by group.

Name of group	No. of Members	Organic rice	Percent	Conventional rice	Percent
- Rice Fund Surin Organic Agriculture Cor.	434	152	33.9	147	36.7
- Nature Care Club	240	105	23.4	98	24.4
- Kaokunda Chaonakunnadham project	147	73	16.3	61	15.2
- Bakruea Farmer Group Network	118	67	15.0	50	12.5
- North Yasothorn Organic Agriculture Pro.	106	51	11.4	45	11.2
Total	1,045	448	100.0	401	100.0
2) Group of conventional rice farmers. This study wanted to evaluate the sentiments of farmers who grow conventional rice, has an area close to organic rice farmers, saw organic farming behaviour, and had not changed to growing organic rice. The population of farmers growing rice close to the five organic rice groups was 2,350 (National Statistical Office, 2016).

Next, the researchers randomly distributed questionnaires to each group: 500 samples were returned, including samples for unresponsive and incomplete data. The number of completed questionnaires from organic rice farmers was 448 samples, and conventional rice farmers was 401 samples that passed the minimum sample requirements of the research (370 samples; Table 1).

## 4 Results

First, the statistical results in Table 2 compares the mean of the two groups (Group 1 = organic farmers, Group 2 = conventional farmers), and the organic rice group had a higher value than the conventional rice group. The highest mean difference between groups was the PBF at = 1.298, followed by AFB at = 1.148, and the PRF at = 1.045. The lowest value was the SGP at = 0.693. In addition, the standard deviation (SD) and coefficient of variation (CV) demonstrated that conventional rice farmers had higher values than organic rice farmers when the information of Group 2 was distributed over that of Group 1. Furthermore, the variance inflation factor (VIF) showed that neither group had multi–collinearity problems, because the VIF was not greater than 10 (Hair et al., 2010). Regarding Cronbach's coefficient, Group 1 had a value ranging between .770 and .908, and Group 2 had a value between .755 and .916. The value of the two research groups was more than 0.70; thus, the collected data were consistent (Pallant, 2007). Likewise, the Pearson's correlation between both groups was less than .800 (Table 3).

**Table 2:** Verification of the validity of the questionnaire.

Variables	G	$\bar{\text{x}}$	MeanDiff.	SD	CV	VIF	Cronbach's Alpha
Attitudes towards Farming Behaviour (AFB)	1	4.468	1.148	.446	.099	1.679	.858
	2	3.320		.747	.225	2.158	.883
Group-Norm Influences on Farming Behaviour (GFB)	1	4.506	0.693	.493	.109	1.957	.894
	2	3.813		.629	.165	2.049	.851
Perceived Behavioural Control of Farmers (PBF)	1	4.282	1.298	.557	.130	2.170	.854
	2	2.984		.907	.304	1.423	.905
Comparative Usefulness of Behaviour (CUB)	1	4.397	0.932	.464	.106	3.237	.803
	2	3.465		.655	.189	2.817	.825
Perception of Risk of Farming (PRF)	1	4.411	1.045	.539	.122	2.399	.898
	2	3.366		.654	.194	1.935	.850
Support of Government Policy (SGP)	1	4.576	0.876	.475	.104	1.468	.908
	2	3.700		.719	.194	2.535	.916
Intention toward Organic Farming (IOF)	1	4.430	1.110	.495	.112	-	.770
	2	3.320		.735	.221	-	.755

**Table 3:** Pearson's correlation coefficients between variables.

Variables	Group	AFBs	GFB	PBF	CUB	PRF	SGP	IFB
AFB	1	1.000						
	2	1.000						
GFB	1	.460**	1.000					
	2	.661**	1.000					
PBF	1	.525**	.590**	1.000				
	2	.379**	.420**	1.000				
CUB	1	.599**	.654**	.687**	1.000			
	2	.589**	.585**	.495**	1.000			
PRF	1	.507**	.605**	.617**	.725**	1.000		
	2	.489**	.466**	.452**	.664**	1.000		
SGP	1	.426**	.395**	.352**	.527**	.481**	1.000	
	2	.626**	.579**	.454**	.725**	.589**	1.000	
IOF	1	.579**	.591**	.545**	.660**	.639**	.539**	1.000
	2	.556**	.563**	.629**	.641**	.534**	.650**	1.000

**p < .01.

Afterwards, confirmatory factor analysis (CFA) was conducted using a statistical programme to compare the two groups (Group 1 = organic, Group2 = conventional). The calculated values for chi-square = 2,454.647 (df = 1,220) differed at a significance level of .05. Accordingly, CFI = .915, TLI = .907, RMSEA = .049, and SRMR = .072. In summary, all values fulfilled the requirements for model fit of SEM, CFI or TLI >0.900, RMSEA <0.070, and SRMR <0.080 (Hair et al., 2006).

**Figure 2:**
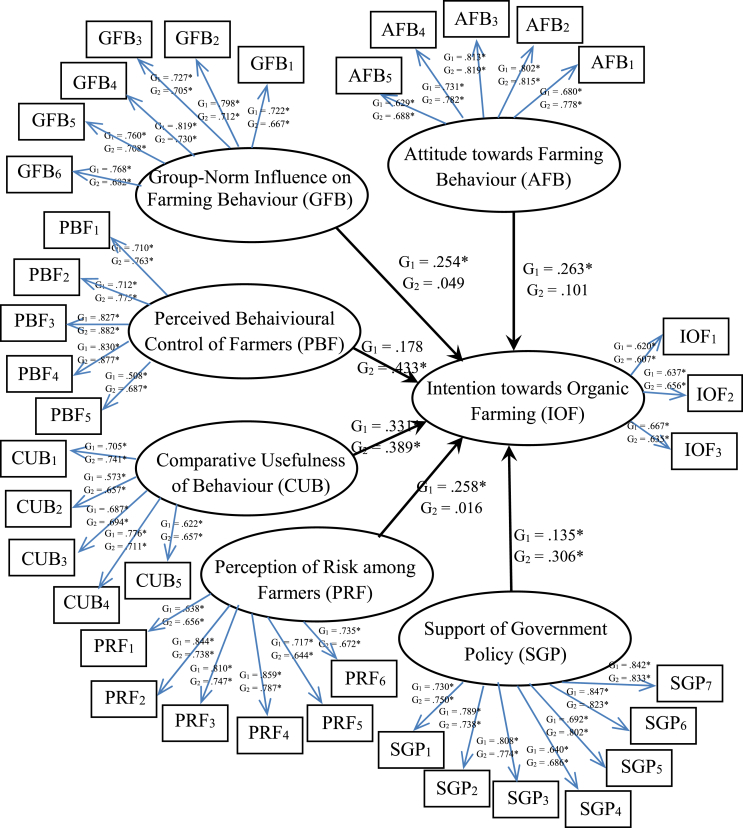
Comparison factors influencing the intention toward organic farming (IOF).

Table 4 compares the factor loadings and R2 values. The lowest and highest values for factor loadings for Group 1were .508 and .859, and for R2 were .307 and .738; the lowest and highest values for Group 2 were .573 and .881, and for R2 were .368 and .778, respectively. As a result, the factor loadings of both groups were higher than the standard set by Hair et al. (2010), who suggested that factor loadings should be greater than .300 and R2 values should be significant. The statistical analyses presented Table 5 and Figure 2 were based on the model of the factors that influenced IOF as follows.

**Table 4:** Comparison of factor loadings and R2 values.

Variables	Group	Factor Loading	R^2^
Attitudes towards Farming Behaviour (AFB)	1	.629* – .813*	.396* – .660*
	2	.688* – .819*	.474* – .671*
Group-Norm Influences on Farming Behaviour (GFB)	1	.722* – .813*	.521* – .637*
	2	.667* – .730*	.445* – .532*
Perceived Behavioural Control of Farmers (PBF)	1	.508* – .830*	.307* – .689*
	2	.689* – .881*	.472* – .778*
Comparative Usefulness of Behaviour (CUB)	1	.573* – .776*	.329* – .602*
	2	.657* – .741*	.431* – .548*
Perception of Risk of Farming (PRF)	1	.638* – .859*	.407* – .738*
	2	.644* – .787*	.431* – .619*
Support of Government Policy (SGP)	1	.640* – .847*	.409* – .717*
	2	.686* – .833*	.470* – .693*
Intention toward Organic Farming (IOF)	1	.620* – .667*	.384* – .445*
	2	.607* – .656*	.368* – .430*

*p < .05.

**Table 5:** Comparison factors influencing the intention toward organic farming (IOF).

Variables	Group	Influence on the IOF	Power to explain IOF, R2	
			Group 1	Group 2
Attitudes towards Farming Behaviour (AFB)	1	.263*	.821*	.854*
	2	.101		
Group-Norm Influence toFarming Behaviour (GFB)	1	.254*		
	2	.049		
Perceived Behavioural Control of Farmers (PBF)	1	.178		
	2	.433*		
Comparative Usefulness of Behaviour (CUB)	1	.332*		
	2	.389*		
Perception of Risk of Farming (PRF)	1	.258*		
	2	.016		
Support of Government Policy (SGP)	1	.135*		
	2	.306*		

*p < .05.

### 4.1 Attitudes towards farming behaviour (AFB)

The results from organic rice farmers (Group1) showed that AFB were significant and positively influenced IOF (β = .263). By contrast, the results from the conventional rice farmers (Group2) showed that AFB were not significant and only slightly positively influenced IOF (β = .101). Thus, attitudes towards farming influenced the growing of organic rice. Group1 recognises the benefits of organic rice that made them have the intention to grow it in their next round of crops. By contrast, Group2 had not yet grown organic rice. The conventional rice farmers (Group2) recognised that organic farming is a good practice but not enough to influence their intentions to grown organic rice. In the same manner, Borges et al. (2016) studied how the attitudes of farmers influenced a farmer's intention to improve grassland (β = .460), and Lalani et al. (2016) found that farmers' attitudes influenced intentions to use conservation agriculture (β = .593). Moreover, farmers' attitudes were influential with regard to intention towards farming behaviour: Jones et al. (2016), β = .497; van Dijk et al. (2016), β = .170; Sok et al. (2016), β = .610; and Deng et al. (2016), β = .327. As a result, regarding the following, organic farmers agree on AFB higher than conventional farmers: 1) the product quality from organic rice farming is better than that from conventional rice farming, 2) organic farming is good for the health of farmers and their family members, 3) the products from organic farming are good for consumers' health, 4) organic farming is good for the environment; and 5) organic farming promotes the well-being of families. If farmers agree that they have positive attitudes towards organic rice cultivation, they will intend to grow organic rice.

### 4.2 Group–norm influences on farming behaviour (GFB)

The results from organic rice farmers (Group1) showed that the GFB was significant and positively influenced IOF (β = .254), whereas the results from conventional rice farmers (Group2) showed that GFB was not statistically significant and had a slightly positive influence on IOF (β = .049). The GFB influenced the intentions of Group1 more than Group2 because organic rice farmers know the advantages of group–membership such as obtaining a high price. By contrast, conventional farmers observed the organic rice groups' behaviour and had conversations about the benefits of organic rice. The conventional rice farmers (Group2) recognised that membership in an organic–group was good, but not enough to influence their intentions to grown organic rice. Likewise, Deng et al. (2016) showed that person/group–norms influenced farming behaviour with regard to payment for the ecosystem service programme (β = .418), and Jones et al. (2016) found that group–norms influenced farmers' intentions to improve herd health (β = .495). In addition, person/group–norms influenced farmers’ intentions towards other behaviours: van Dijk et al. (2016), β = .110; Sok et al. (2016), β = .180; Borges et al., (2016), β = .237; and Dang et al. (2014), β = .118. Therefore, regarding the following, organic farmers agree on GFB higher than conventional farmers: 1) organic farmer membership is positive, 2) organic farmer membership is positive for the organics certificate, 3) membership in the organic farmer group engenders credibility to the rice–export market, 4) organic farmer membership increases the exchange of information on products and marketing, 5) organic farmer membership strengthens cooperation in the group, and 6) organic farmer membership has increased group awareness. Finally, if farmers agree that membership in the organic rice farmer group is positive, the intent to grow organic rice will increase.

### 4.3 Perceived behavioural control of farmers (PBF)

The results from organic rice farmers (Group1) showed that the PBF was not significant and had a slight positive influence on IOF (β = .178), whereas the results from conventional rice farmers (Group2) showed that the PBF was significant and had the highest positive influence on IOF (β = .433). PBF influenced the intentions of Group 2 more than Group1 because conventional farmers focus on knowledge, techniques, and methods of organic rice cultivation. If the self-evaluation of conventional rice farmers on PBF is sufficient for growing organic rice, they will change and begin grow it. By contrast, organic rice farmers have experience and are members of a strong group that received certified organic standards. They appreciate that PBF is not a critical issue in the intention to growing organic rice when compared with other factors. Similarly, Jones et al. (2016) found that the PBF had a strong influence on the intention to improve herd health (β = .523); Lalani et al. (2016) found that the PBF influenced the intention to use conservation agriculture (β = .341). Moreover, several studies have confirmed the perceived ability of farmers to control their behaviours: Deng et al. (2016), β = .496; van Dijk et al. (2016), β = .120; and Borges et al. (2016), β = .218. As a result, conventional farmers’ concern about the PBF is higher than that of organic farmers. Therefore, if conventional farmers evaluate themselves on this issue: 1) able to control the expected yield of organic rice, 2) might grow rice in accordance with organic standards, 3) knowledge of the techniques and methods of planting non–toxic rice, 4) be confident that their knowledge regarding organic rice cultivation, and 5) be confident that their rice would be certified as organic. Finally, if a farmer evaluates the PBF as appropriate for organic rice, they will intend to grow it.

### 4.4 Comparative usefulness of behaviour (CUB)

The results from organic rice farmers (Group1) showed that CUB was significant and had a high positive influence on IOF (β = .332). Likewise, the results from conventional rice farmers (Group2) showed that the CUB was significant and had a high positive influence on IOF (β = .389). The CUB influenced the intentions of Group 1 and Group 2 because the farmers’ perception of the utility of organic rice was more than that of conventional rice. Consequently, the CUB will influence farmers to grow organic rice. By contrast, Aubert et al. (2012), Sattler and Nagel (2010), and Warren et al. (2016) have found that farmers acknowledge the benefits of behaviours that promote the intention to adoption behaviours. In this research, the β-value was relatively high in both groups, which shows that the farmers viewed the following aspects of CUB as important: 1) organic rice planting is good for the ecosystem and soil fertilisation compared with conventional rice planting, 2) organic rice farmers are more diligent than conventional rice farmers, 3) organic rice is more expensive than conventional rice, 4) organic farming costs less than conventional farming because fertilizers and pesticides are not used, and 5) organic farming uses the same equipment and machinery in the same manner as conventional farming. Finally, if farmers compare organic farming with conventional farming and consider organic farming better, they will intend to grow organic rice.

### 4.5 Perception of the risk of farming (PRF)

The results from organic rice farmers (Group 1) showed that the PRF was significant and had a positive influence on IOF (β = .258), whereas the results from conventional rice farmers (Group 2) showed that the PRF was not significant and had a slight positive influence on IOF (β = .016). The PRF influenced intentions of organic rice farmers more than conventional rice farmers because they have experience with both types of rice. Therefore, organic farmers know the hazards of agricultural chemicals and are committed to growing organic rice. By contrast, conventional rice farmers with no experience in growing organic rice knew the hazards of agricultural chemicals used in growing conventional rice, this did not have enough influence to encourage farmers to grow organic rice. Yazdanpanah et al. (2014) found that the risk perceptions of farmers affected the intention to practice water conservation in agriculture (β = .140), and Dang et al. (2014) found that the risk perceptions of farmers about how climate change affects productivity, financial status, and health influenced intentions to accommodate changing weather conditions (β = .155). As a result, organic farmers agree on PRF issue higher than conventional farmers. Therefore, they have the following perceptions of risk on this issue: 1) increases the agricultural cost of fertilisers and pesticides, 2) families members could be exposed to hazards from the use of fertilisers and pesticides; 3) growers will be harmed by using fertilisers and pesticides, 4) the long-term use of fertilisers and pesticides will increase every year, 5) conventional rice could exceed the market demand, and 6) lower pricing of conventional rice will decrease incomes. Finally, if farmers are aware of the PRF associated with conventional rice farming, then they will intend to grow organic rice.

### 4.6 Support of government policy (SGP)

The results from organic rice farmers showed that the SGP was significant and positively influenced IOF (β = .135). Similarly, the results from the conventional rice farmers showed that the SGP was significant and had a high positive influence on IOF (β = .306). Other findings support this finding, for example, Tate et al. (2012) found that government and local council support affected farmers’ adoption of renewable energy (β = .014), and investment credits influenced the application of renewable energy (β = .016). By contrast, Dang et al. (2014) found that policy did not affect intention to purchase crop insurance because of climate change. In addition, SGP is the factor that was significant for both organic and conventional rice farmers. The reason that SGP is affected is because the policies meets the needs of small-scale farmers who lack the knowledge, equipment, technology, and financial resources for farming. It is critical for smallholder farmers to affect the IOF. Therefore, the farmers awareness on this issue is as followa: Government policy should support 1) irrigation efforts for organic rice farming (e.g. digging ponds, wells, pumping stations), 2) the certification of the prices of organic rice, 3) organic rice exports, 4) production equipment acquisition (e.g., seed, organic fertiliser, and organic rice mills), 5) low-interest loans for organic rice farmers; 6) assistance for farmers to certify organic rice standards, and 7) cultivation knowledge and techniques to increase the productivity of organic rice. Finally, if a farmer evaluates the SGP as positive to organic rice, they will intend to grow it.

## 5 Recommendations and further reasearch

This section is an outline of how to increase organic farming, especially smallholder farmers, by establishing effective policies that support them. Regarding the AFB, the conventional farmers contunue to have a negative attitude towards organic rice farming. Thus, government should implement a policy to increase understanding and promote the benefits of organic rice farming, and increase the awareness of the risks and dangers of conventional agriculture behaviour. In addition, regarding the group–norm influences on farming behaviour, the government should have a policy to encourage conventional farmers to join the organic rice member–group and to promote the knowledge on and techniques for growing certified organic rice to help farmers with their perceived behavioural control, which would promote the intention to grow organic rice. As a result, government agencies should apply policies with more incentives for farmers change to organic farming.

### 5.1 Two categories of short-term policies

#### 5.1.1 Organic farmers

Policy should focus on encouraging farmers to grow organic rice and encourage farmers to join the organic group. Government policies should support equipment and input factors for the organic group, such as sources of water, machinery for preparation and harvesting, and low–interest funding. Therefore, government policies to strengthen the organic group that incentivise non–participating farmers to join the organic farming group and motivate older–members to continue with the group are necessary.

#### 5.1.2 Conventional farmers

Policies should focus on motivating farmers to change their behaviours towards organic farming and encourage membership in the organic farmers group. In particular, a focus should be on public relations to increase the understanding of organic agriculture, including field trips to successful organic farms. Furthermore, policies should encourage farmers to switch to organic farming through support for, for example, equipment, irrigation, low–interest capital for switching to organic farming, and organic farming certification.

### 5.2 Long-term policies should focus on the sustainability of organic farming

Therefore, farmers should be encouraged to continuously use organic farming methods and not switch to chemical farming. The government should continue to promote the benefits of organic cultivation and encourage continuous and expansive support of the organic farmer group. Afterwards, the support for the organic farmers group will be strong, and the group might be self–sufficient through knowledge, technology, and the production of organic plants of high quality and high yield. In addition, organic farmers might support themselves through knowledge, technology, and training based on the creation and innovation of new products to add value to organic raw materials that farmers could sell at a high price.

The application of the IOF model in further research should compare three groups: organic rice farmers (Group1), conventional rice farmers growing rice close to organic rice groups (Group2), and conventional rice farmers growing rice in remote areas away from organic rice groups (Group3). The outcome will lead to comprehensive policies that expand organic agriculture.

## Declarations

### Author contribution statement

P. Yanakittkul: Conceived and designed the experiments; Performed the experiments; Analyzed and interpreted the data; Contributed reagents, materials, analysis tools or data; Wrote the paper.

C. Aungvaravong: Conceived and designed the experiments; Analyzed and interpreted the data; Contributed reagents, materials, analysis tools or data; Wrote the paper.

### Funding statement

This research did not receive any specific grant from funding agencies in the public, commercial, or not-for-profit sectors.

### Competing interest statement

The authors declare no conflict of interest.

### Additional information

No additional information is available for this paper.
