# Supplementary material for: A model of farmers intentions towards organic farming: A case study on rice farming in Thailand
Source: Heliyon. 2019 Dec 28;6(1):e03039. doi: 10.1016/j.heliyon.2019.e03039 (PMC6939084; doi:10.1016/j.heliyon.2019.e03039)
Supplement: supplementary material [file mmc1.doc]

**Appendix A.**

The questionnaires used to Likert-scales with five levels: (1) very low, (2) low, (3) moderate, (4) high, and (5) very high

| Latent Variable | Observation Variables | 1 | 2 | 3 | 4 | 5 |
| --- | --- | --- | --- | --- | --- | --- |
| Attitude towards Farming Behaviour (AFB) | - Quality of product from organic farming is better than conventional farming. (AFB_1_)  - Organic farming is good for farmers and the health of family members. (AFB_2_)  - The products from organic farming are good for consumers’ health. (AFB_3_)  - The products from organic farming are good for the environment. (AFB_4_)  - Organic farming will promote the well-being of families. (AFB_5_) |  |  |  |  |  |
| Group-Norm Influence on Farming Behaviour (GFB) | - Organic farmer membership is positive. (GFB_1_)  - Organic farmer membership is positive for the organics certificate. (GFB_2_)  - Membership in the organic farmer group engenders credibility to the rice–export market. (GFB_3_)  - Organic farmer membership increases the exchange of information on products and marketing. (GFB_4_)  - Organic farmer membership strengthens cooperation in the group. (GFB_5_)  - Organic farmer membership has increased group awareness. (GFB_6_) |  |  |  |  |  |
| Perceived Behaivioural Control of Farmers (PBF) | - Able to control the expected yield of organic rice. (PBF_1_)  - Might grow rice in accordance with organic standards. (PBF_2_)  - Knowledge of the techniques and methods of planting non–toxic rice. (PBF_3_)  - Be confident that their knowledge regarding organic rice cultivation. (PBF_4_)  - Be confident that their rice would be certified as organic. (PBF_5_) |  |  |  |  |  |
| Comparative Usefulness of Behaviour (CUB) | - Organic rice planting is good for the ecosystem and soil fertilization compared with conventional rice planting. (CUB_1_)  - Organic rice farmers are more diligent than conventional rice farmers. (CUB_2_)  - Organic rice is more expensive than conventional rice. (CUB_3_)  - Organic farming costs less than conventional farming because fertilizers and pesticides are not used. (CUB_4_)  - Organic farming uses the same equipment and machinery in the same manner as conventional farming. (CUB_5_) |  |  |  |  |  |
| Perception of Risk among Farmers (PRF) | - Increases the agricultural cost of fertilisers and pesticides. (PRF_1_)  - Families members could be exposed to hazards from the use of fertilisers and pesticides. (PRF_2_)  - Growers will be harmed by using fertilisers and pesticides. (PRF_3_)  - The long-term use of fertilisers and pesticides will increase every year (PRF_4_)  - Conventional rice could exceed the market demand. (PRF_5_)  - Lower pricing of conventional rice will decrease incomes (PRF_6_) |  |  |  |  |  |
| Support of Government Policy (SGP) | - Support irrigation efforts for organic rice farming (e.g. digging ponds, wells, pumping stations). (SGP_1_)  - Support the certification of the prices of organic rice. (SGP_2_)  - Support organic rice exports. (SGP_3_)  - Support production equipment acquisition (e.g., seed, organic fertiliser, and organic rice mills). (SGP_4_)  - Support low–interest loans for organic rice farmers. (SGP_5_)  - Support assistance for farmers to certify organic rice standards. (SGP_6_)  - Support cultivation knowledge and techniques to increase the productivity of organic rice. (SGP_7_) |  |  |  |  |  |
| Intention towards Organic Farming (IOF) | - Farmers intend to organic farming. (IOF_1_)  - Farmers intend to participate with activities to promote organic farming. (IOF_2_)  - Farmers are interested in learning and searching for knowledge about organic farming. (IOF_3_) |  |  |  |  |  |
